# Efficacy and mechanisms of low-frequency TENS for common multimorbidity in older adults: protocol for a pilot randomized controlled trial

**DOI:** 10.3389/fmed.2026.1788268

**Published:** 2026-04-22

**Authors:** An Yu, Liuyan Wang, Xi Chen, Mei Xie, Bijuan Liang, Qi Wu, Xiang Li, Ying Gao, Lei Yang

**Affiliations:** 1 Yunnan Key Laboratory of Cell Therapy for Refractory Diseases, Kunming University, Kunming, China; 2 Department of Rehabilitation Medicine, The Second People’s Hospital of Kunming, Kunming, Yunnan, China; 3 Department of Geriatrics, The Second People’s Hospital of Kunming, Kunming, Yunnan, China

**Keywords:** essential hypertension, hyperlipidemia, multimorbidity, randomized controlled trial, study protocol, transcutaneous electrical nerve stimulation, type 2 diabetes mellitus

## Abstract

**Background:**

Multimorbidity (co-occurrence of ≥2 chronic conditions) in older adults is a global health challenge, often managed with polypharmacy that carries risks of adverse events and drug–drug interactions. As a pilot randomized controlled trial (RCT), this study first aims to assess the feasibility of low-frequency transcutaneous electrical nerve stimulation (Lo-TENS) delivery in older adults with multimorbidity and estimate effect sizes for a subsequent definitive trial. Second, we examine the efficacy of Lo-TENS as an adjunct to routine clinical management for improving blood pressure, glycemic control, and lipid profiles, and explore the underlying autonomic nervous system (ANS) modulation mechanisms through analysis of heart rate variability and key inflammatory/metabolic biomarkers, which are hypothesized to mediate Lo-TENS’s beneficial effects on clinical indicators.

**Methods:**

A pilot single-center, single-blinded, sham-controlled RCT will enroll 50 older adults (≥60 years) with multimorbidity (≥2 of EH, T2DM, hyperlipidemia). Participants will be randomly allocated (1,1) to receive Lo-TENS (2 Hz, 30 min/day, 5 days/week for 4 weeks) plus routine care or sham Lo-TENS plus routine care. Co-primary outcomes are office systolic blood pressure (SBP), fasting blood glucose (FBG), and low-density lipoprotein cholesterol (LDL-C) at post-intervention. Secondary outcomes include 24-h ambulatory BP, heart rate variability (HRV), inflammatory/metabolic biomarkers, and quality of life. Mechanistic analyses will explore mediation by ANS and inflammatory markers. Follow-up assessments will be conducted 4 weeks post-intervention.

**Discussion:**

This trial will provide evidence on the effectiveness of Lo-TENS on clinical outcomes in multimorbidity in older adults, and explore the underlying mechanisms through which Lo-TENS may alleviate the clinical indicators of multimorbidity, specifically by analyzing heart rate variability and key inflammatory and metabolic biomarkers.

**Trial registration:**

www.chictr.org.cn, identifier ChiCTR2400093956.

## 1 Introduction

Multimorbidity, defined as the co-occurrence of two or more chronic conditions in a single individual, affects over 60% of adults aged ≥60 years globally and is associated with increased mortality, reduced quality of life, and high healthcare costs (1, 2). Common multimorbidity combinations in older adults include essential hypertension (EH), type 2 diabetes mellitus (T2DM), and hyperlipidemia—conditions that share overlapping pathophysiological pathways and often require polypharmacy for management (3). While polypharmacy is a cornerstone of multimorbidity care, it carries significant risks in older adults, including drug–drug interactions, adverse drug events, and reduced treatment adherence (4, 5), highlighting an urgent need for safe, non-pharmacological adjunctive therapies (6, 7).

Sympathetic over-activation has emerged as a unifying pathophysiological driver of EH, T2DM, and hyperlipidemia, particularly in older adults (8, 9). Age-related declines in autonomic nervous system (ANS) regulation lead to increased sympathetic tone, which exacerbates vascular dysfunction (contributing to EH), impairs insulin sensitivity (worsening T2DM), and promotes pro-inflammatory responses (driving hyperlipidemia) (10, 11). In older adults with multimorbidity, sympathetic over-activation exerts synergistic adverse effects across cardiovascular and metabolic systems: increased catecholamine release elevates vascular tone (EH) and impairs insulin sensitivity (T2DM), while sympathetic-driven pro-inflammatory responses exacerbate lipid dysregulation (hyperlipidemia) (12). This unifying pathophysiology creates a unique therapeutic target—ANS modulation—for a single adjunctive intervention to improve multiple co-existing conditions simultaneously (13).

However, no pilot randomized controlled trial (RCT) has yet investigated Lo-TENS for common multimorbidity in older adults, nor explored its mechanistic pathways through ANS and inflammatory/metabolic biomarkers. Low-frequency transcutaneous electrical nerve stimulation (Lo-TENS; ≤10 Hz) is a non-invasive, low-risk intervention that modulates ANS activity by stimulating peripheral nerves (14). Prior studies have shown that Lo-TENS reduces sympathetic tone (measured via plasma norepinephrine levels and HRV) and improves clinical outcomes in patients with single chronic conditions, such as EH and T2DM (15–17) For example, Stein et al. demonstrated that 2 Hz Lo-TENS robustly reduces sympathetic activity and increases parasympathetic tone in healthy adults (15), and subsequent studies have validated these effects in older adults with cardiovascular conditions (18, 19). A recent randomized trial in patients with type 2 diabetes further suggested that TENS may improve glycemic variability, although its effect on HbA1c remains inconclusive, warranting further mechanistic investigation (20).

A pilot RCT is critical for this research question to assess feasibility (e.g., recruitment, intervention adherence, attrition) and estimate effect sizes for a subsequent definitive trial (21). As an pilot RCT, this study first aims to assess the feasibility of Lo-TENS delivery in older adults with multimorbidity and estimate effect sizes for a subsequent definitive trial. Second, we examine the efficacy of Lo-TENS as an adjunct to routine clinical management for improving blood pressure, glycemic control, and lipid profiles in older adults with common multimorbidity (≥2 of EH, T2DM, hyperlipidemia). Third, we explore the underlying mechanisms of Lo-TENS by testing whether changes in ANS function (HRV, norepinephrine) and inflammatory/metabolic biomarkers (IL-6, adiponectin, FGF21) mediate its effects on clinical outcomes (22, 23).

## 2 Methods

### 2.1 Study design

**Figure 1:**
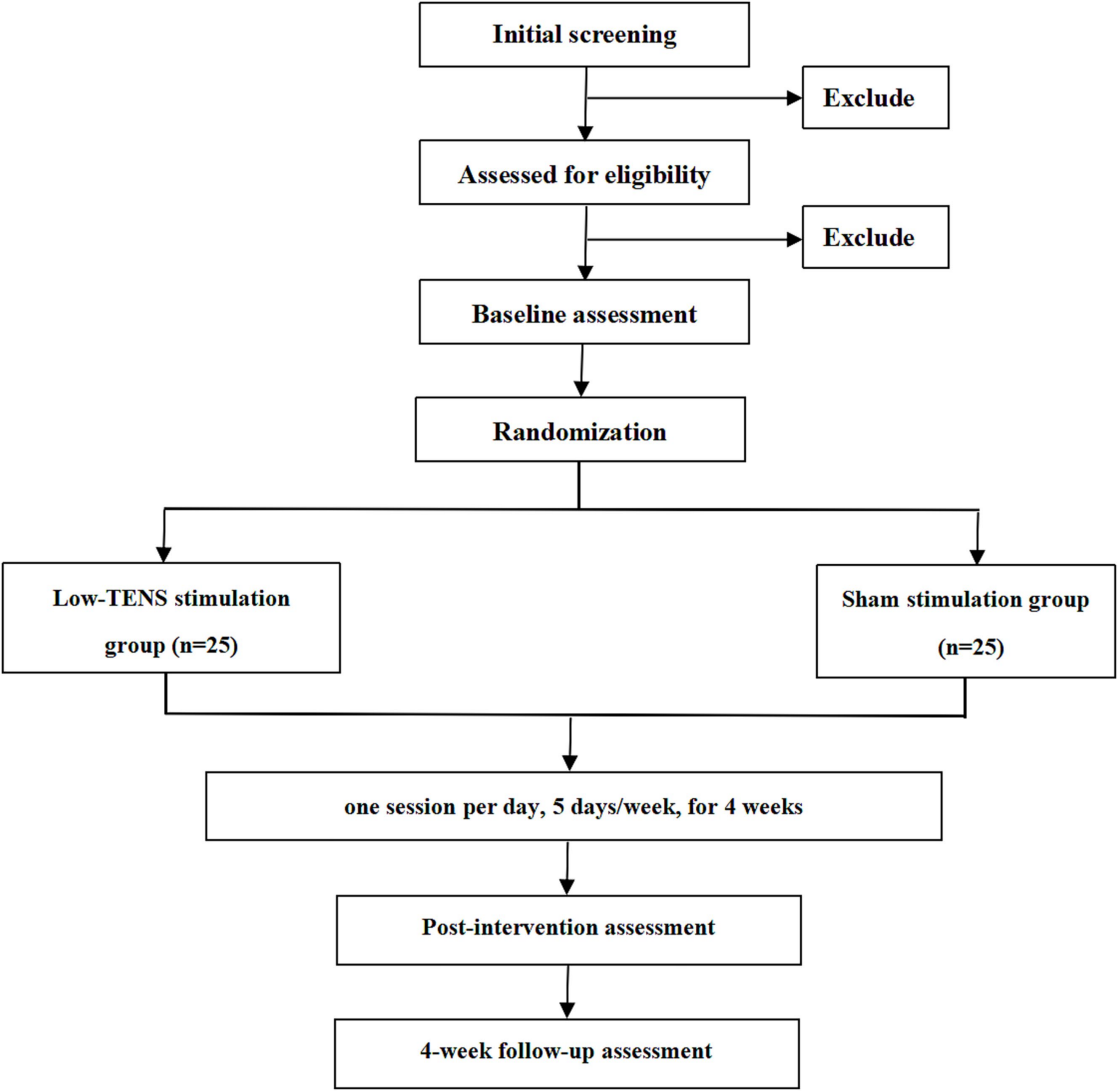
Study flowchart of trial process.

This is a single-center, single-blinded, sham-controlled pilot RCT conducted at The Second People’s Hospital of Kunming (Kunming, China). The study adheres to the SPIRIT (Standard Protocol Items: Recommendations for Interventional Trials) guidelines (24) and TIDieR (Template for Intervention Description and Replication) checklist (25) (see Table 1). Participants will be randomly allocated to either the Lo-TENS group or the sham Lo-TENS group in a 1:1 ratio. The intervention period is 4 weeks, with follow-up assessments conducted 4 weeks post-intervention (total study duration: 8 weeks per participant). The study flowchart is presented in Figure 1.

**Table 1:** The intervention protocol for each group base on the TIDieR template.

Item	1. Lo-TENS plus routine clinical management group	2. Sham stimulation plus routine clinical management group
1. Why	The stimulation protocol is adopted from previous studies. A self-adhesive electrode plate is affixed to the dorsal aspect of the first and second metacarpal bones, an additional electrode plate is positioned over the muscle belly of the proximal radius, with both upper limbs receiving the same placement (Figure 2). Therefore, the same protocol was adopted in our trial. The routine clinical management is to ensure participants’ multimorbidity is stable.	The sham stimulation group served as an active control group, enabling us to determine whether the observed changes in the stimulation groups are a function of maturation, repeated testing, or placebo effect.
2. What materials	KD-2A, Yaoyang Kangda Electronic Instrument Co., Ltd., Beijing, China. 5 × 5 cm self-adhesive electrodes.	KD-2A, Yaoyang Kangda Electronic Instrument Co., Ltd., Beijing, China. 5 × 5 cm self-adhesive electrodes.
3. Procedures	Participants will be sitting in a comfortable chair. After cleaning the skin, Lo-TENS therapy will be performed. One channel to one upper limb, and both upper limbs will be placed.	The placement of electrodes is the same as in group 1.
4. Who provided	Experienced physiotherapists	Experienced physiotherapists
5. How	The stimulation parameters are set as follows: mode, continuous; waveform, bidirectional asymmetric square wave; pulse width, 200 μs; frequency, 2 Hz. The stimulation intensity in milliamps (mA) will be tailored to the individual’s sensation, permitting slight muscle contraction but ensuring that pain or any discomfort is avoided.	Except there will be no actual treatment, the other setting will be the same as in group 1.
6. Where	The therapy will be performed in the physiotherapy room of the hospitals.	The therapy will be performed in another physiotherapy room of the hospitals.
7. When and how much	Participants in this group will undergo their stimulation in one 30-min session per day, over a period of 5 days per week, for a total of 4 consecutive weeks (resulting in 20 sessions in total).	Except there will be no actual treatment, the others will be the same as in group 1.
8. Tailoring	The stimulating intensity will be tailored to the participant’s tolerance, but without any pain or discomfort.	As no electrical current is delivered, individualized adjustment of stimulation intensity is not applicable. However, participants may adjust their posture to ensure comfort.
9. How well	Intervention adherence will be quantified as the percentage of completed sessions (≥80% adherence defined as successful); assessors are blinded to group allocation to ensure objective outcome measurement.	Same as Lo-TENS group; adherence is quantified as session completion, and assessor blinding is maintained for objective outcome measurement.

### 2.2 Participants

#### 2.2.1 Eligibility criteria

Inclusion criteria: (1) Aged ≥60 years; (2) Diagnosis of multimorbidity, defined as co-occurrence of ≥2 of the following: EH (diagnosed per Chinese Guidelines for the Management of Hypertension (26)), T2DM (diagnosed per American Diabetes Association guidelines (27)), hyperlipidemia (diagnosed per Chinese Guidelines for the Management of Dyslipidemia in Adults (28)); (3) Medically stable, defined as no acute exacerbation of EH, T2DM, or hyperlipidemia in the past 3 months and no hospitalizations for cardiovascular/metabolic events (e.g., myocardial infarction, stroke, diabetic ketoacidosis) in the past 6 months; (4) Able to understand and comply with the intervention protocol (e.g., attend 4 weeks of TENS sessions); (5) Voluntary participation and signed informed consent.

Exclusion criteria: (1) Contraindications to TENS (e.g., pacemaker/implantable cardioverter-defibrillator, skin lesions at electrode sites, pregnancy); (2) Severe cognitive impairment (Mini-Mental State Examination score <24); (3) Severe comorbidities (e.g., end-stage renal disease, malignant tumors); (4) Use of other non-pharmacological interventions for cardiovascular/metabolic conditions (e.g., acupuncture, massage) within the past 1 month; (5) Unstable medication regimens for EH/T2DM/hyperlipidemia (e.g., dose changes within the past 2 months).

#### 2.2.2 Recruitment and informed consent

Participants will be recruited from the outpatient department and community health centers of The Second People’s Hospital of Kunming between December 2025 and May 2027. Recruitment strategies include poster advertisements, physician referrals, and community health education sessions. Eligible participants will be invited to attend a pre-recruitment briefing, where study staff will explain the study purpose, procedures, risks, and benefits. Those who agree to participate will sign a written informed consent form (approved by the hospital’s Ethics Committee) prior to any study-related procedures. Informed consent will be administered by trained rehabilitation therapists to ensure participants fully understand the protocol.

### 2.3 Interventions

#### 2.3.1 Lo-TENS group

Participants in the Lo-TENS group will receive Lo-TENS plus routine clinical care. Routine care includes standard pharmacological treatment (antihypertensives, antidiabetics, lipid-lowering drugs) and lifestyle advice (diet, exercise) per clinical guidelines, with no changes to medication regimens during the study period.

**Figure 2:**
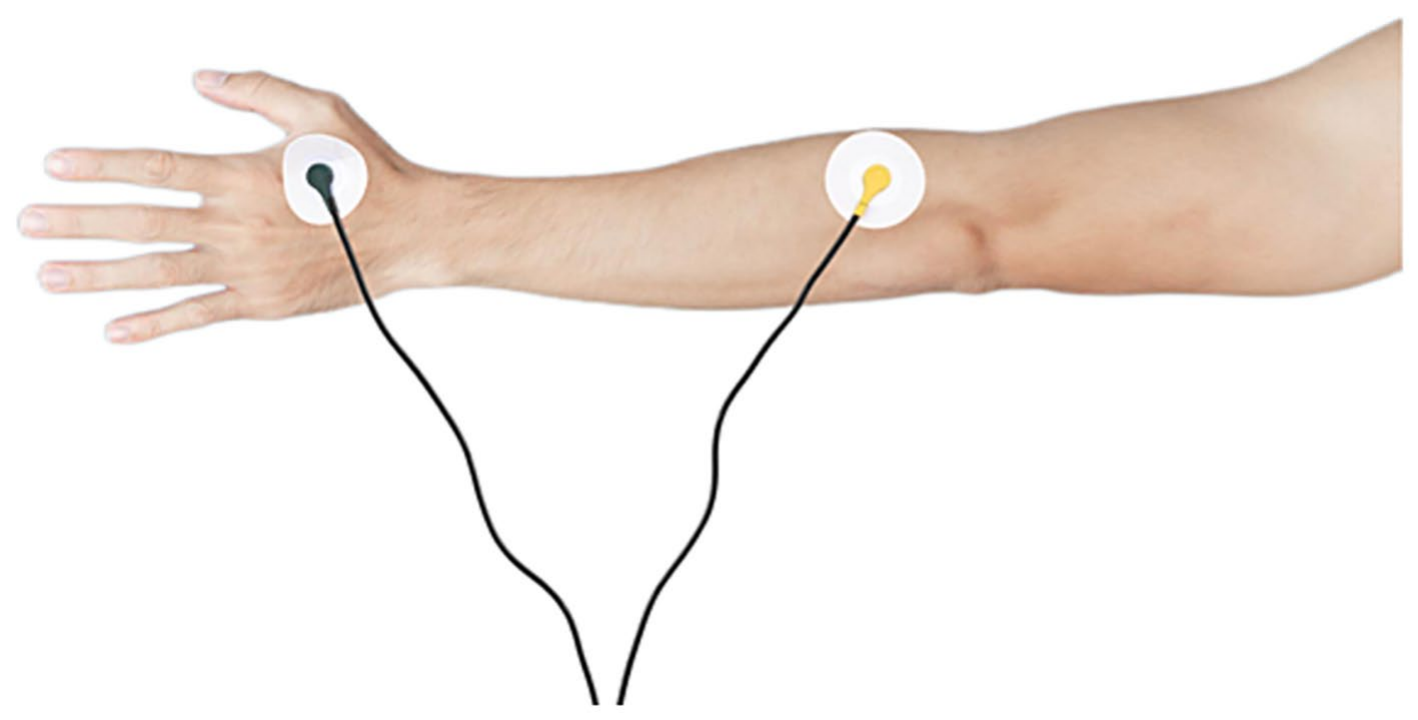
The placement of electrodes.

Lo-TENS will be delivered using a KD-2A TENS device (Beijing Yaoyang Kangda Medical Equipment Co., Ltd., China) with disposable self-adhesive electrodes (5 cm × 7 cm). A 2 Hz frequency was selected based on Stein et al., which demonstrated the most robust reduction in sympathetic activity and increase in parasympathetic tone in healthy adults (7); this frequency has also been validated in prior Lo-TENS studies for cardiovascular/metabolic conditions and is well-tolerated in older adults due to minimal muscle contraction (8, 9). Electrode placement on the dorsal metacarpal bones (1st/2nd) and proximal radius muscle belly targets the median/radial nerves (Figure 2), which project to the spinal cord and brainstem regions regulating ANS activity; this placement avoids fragile skin on the trunk/limb extremities (common in older adults) and is easily accessible for clinical delivery (29). The stimulation parameters are: frequency = 2 Hz, pulse width = 200 μs, intensity = 2–5 mA (adjusted to the participant’s maximum tolerable level without pain). Each session lasts 30 min, 5 days per week, for 4 weeks (total 20 sessions).

The KD-2A Lo-TENS device will be calibrated weekly by a certified biomedical engineer to ensure consistent stimulation parameters (frequency, pulse width, intensity); all devices will be checked for electrode conductivity prior to each session, and defective electrodes will be replaced immediately.

#### 2.3.2 Sham Lo-TENS group

Participants in the sham group will receive sham Lo-TENS plus routine clinical care (identical to the Lo-TENS group). Sham stimulation uses the same KD-2A device and electrode placement, but the device will be turned off after 10 s of stimulation (to mimic the initial “tingling” sensation of real TENS, ensuring blinding) (30). Participants will remain seated for 30 min per session (matching the Lo-TENS group), 5 days per week, for 4 weeks. Device calibration and electrode checks will be identical to the Lo-TENS group to maintain blinding.

#### 2.3.3 Blinding

The study is single-blinded: participants are blinded to group allocation, but therapists (who deliver the intervention) cannot be blinded due to the need to adjust TENS intensity. To minimize bias, outcome assessors and data analysts are also blinded to group allocation. A post-intervention blinding effectiveness survey will be administered to all participants (a 5-point Likert scale: 1 = certain sham, 2 = likely sham, 3 = unsure, 4 = likely real, 5 = certain real). The proportion of participants who correctly guess their group allocation will be calculated to quantify unblinding bias, and results will be reported in the trial’s primary analysis.

### 2.4 Outcomes

All outcomes are measured at baseline, post-intervention (week 4), and 4-week follow-up (week 8) (Table 2).

**Table 2:** Timeline for participants’ enrolment, interventions, and assessments.

Time point	Study period							
	Enrolment	Allocation	Intervention					Close-out (follow-up)
	1 week before	0	1st week	2nd week	3rd week	4th week	Post-intervention	8th week
ENROLMENT								
Eligibility screen	X							
Informed consent	X							
Randomization		X						
INTERVENTIONS:								
Lo-TENS + routine clinical management			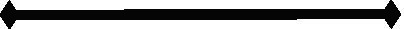					
Sham stimulation + routine clinical management			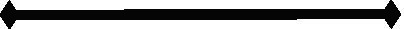					
ASSESSMENTS:								
24-h ABPM	X						X	X
FBG	X						X	X
TC	X						X	X
TG	X						X	X
Plasma glucose	X						X	X
HbA1c	X						X	X
HDL	X						X	X
LDL	X						X	X
LDL-cholesterol	X						X	X
24-h dynamic ECG	X						X	X
CRP	X						X	X
Adiponectin	X						X	X
TNF-α	X						X	X
FGF21	X						X	X
Norepinephrine	X						X	X

ABPM, ambulatory blood pressure monitor (the average, standard deviation, maximum and minimum values of patient’s daytime, nighttime, and all-day systolic, diastolic blood pressure, mean arterial pressure, and pulse pressure); CRP, C-reactive protein; ECG, electrocardiogram; FBG, fasting blood glucose; FGF21, fibroblast growth factor 21; HbA1c, glycosylated hemoglobin; HDL, high-density lipoprotein cholesterol; LDL, low-density lipoprotein; LDL-cholesterol, low-density lipoprotein cholesterol; TC, total cholesterol; TG, triglyceride; TNF-α, tumor necrosis factor-α.

#### 2.4.1 Primary outcomes

Office blood pressure: Systolic blood pressure (SBP) and diastolic blood pressure (DBP) will be measured using a validated electronic sphygmomanometer (Omron HEM-7121) after 5 min of rest; average of 3 measurements (1 min apart).

Fasting blood glucose (FBG): Venous blood sample (8-h fast) measured via enzymatic assay (Beckman Coulter AU5800).

Low-density lipoprotein cholesterol (LDL-C): Measured via direct enzymatic assay to ensure accuracy in participants with hypertriglyceridemia, a common comorbidity in this population; venous blood sample (8-h fast) analyzed using Beckman Coulter AU5800.

#### 2.4.2 Secondary outcomes

24-h ambulatory blood pressure (ABPM): Measured using a Spacelabs 90,217 device (15-min intervals during the day, 30-min intervals at night); primary metrics: 24-h average SBP/DBP, daytime (6:00–22:00) average SBP/DBP, nighttime (22:00–6:00) average SBP/DBP.

Heart rate variability (HRV): Measured via 5-min resting electrocardiogram (ECG) (Philips PageWriter TC50); metrics: time-domain (SDNN, RMSSD) and frequency-domain (HF-HRV, LF-HRV, LF/HF ratio) parameters, analyzed using Kubios HRV software (v3.5) (18, 31).

Inflammatory/metabolic biomarkers: Venous blood samples (8-h fast) analyzed for plasma norepinephrine (NE; enzyme-linked immunosorbent assay [ELISA], R&D Systems), interleukin-6 (IL-6; ELISA, R&D Systems), adiponectin (ELISA, R&D Systems), fibroblast growth factor 21 (FGF21; ELISA, Abcam), and C-reactive protein (CRP; immunoturbidimetry, Beckman Coulter AU5800) (22).

Quality of life (QoL): Assessed using the 36-Item Short Form Health Survey (SF-36); scores range from 0 to 100, with higher scores indicating better QoL.

#### 2.4.3 Feasibility outcomes (pilot-specific)

Feasibility outcomes include: (1) Recruitment rate (number of eligible participants recruited per month); (2) Intervention adherence rate (percentage of participants who complete ≥80% of TENS/sham sessions); (3) Attrition rate (percentage of participants who drop out before post-intervention/follow-up); (4) Adverse event rate (number of adverse events related to TENS/sham stimulation) (21).

### 2.5 Sample size calculation

Sample size calculation is based on the primary outcome of FBG, using G*Power 3.1 software. A 2 × 3 analysis of variance (ANOVA) was selected for sample size calculation due to its suitability for assessing group (Lo-TENS vs. sham) × time (baseline, post-intervention, 4-week follow-up) interactions for continuous primary outcomes; mixed-effects models will be used for secondary analyses to account for potential missing data, consistent with modern pilot trial statistical practices. Based on prior Lo-TENS studies in T2DM (*f* = 0.282, the most conservative effect size) (20), *α* = 0.05, power = 0.80, and a 20% attrition rate, a total sample size of 50 participants (25 per group) is required. This sample size is appropriate for a pilot RCT to estimate effect sizes and assess feasibility.

### 2.6 Randomization and allocation concealment

Randomization sequence is generated using the Random Allocation Software (version 2.0), a validated online tool. Participants are allocated to groups in a 1:1 ratio using permuted blocks of size 4 (to ensure balanced group sizes). Allocation concealment is achieved using opaque, sealed envelopes labeled with sequential participant numbers; envelopes are opened by a researcher not involved in participant recruitment or outcome assessment after informed consent is obtained.

### 2.7 Data collection and management

Data are collected by trained research assistants (blinded to group allocation) at baseline, post-intervention, and 4-week follow-up. Clinical data (BP, FBG, LDL-C) are recorded directly into a secure electronic database (EpiData 3.1). Blood samples are stored at −80 °C until batch analysis to ensure consistency. Dual independent data entry is performed to minimize errors; discrepancies are resolved by a third researcher. All data are anonymized (identified by participant ID only) and stored in a password-protected server at The Second People’s Hospital of Kunming. Data access is restricted to the study team, with the corresponding author responsible for data sharing (per reasonable requests from other researchers).

### 2.8 Statistical analysis

The data analysis will be conducted using the Statistical Package for Social Sciences (SPSS) version 26. To ensure the accuracy of the data entry, it will be performed by two independent researchers who will cross-verify the input data for consistency. The baseline characteristics will be assessed using appropriated statistical tests based on the data distribution: independent *t*-tests for normally distributed data, Mann–Whitney *U* tests for non-parametric data, and Chi-square tests for categorical variables.

If the parameters meet the criteria for normal distribution, a repeated-measures ANOVA will be used to examine group × time interactions for primary and secondary outcomes. To account for multiple comparisons across the three co-primary outcomes (office BP, FBG, LDL-C), a Bonferroni correction will be applied, setting the significance level at *p* < 0.0167. Any two of these outcomes reach significance will be considered positive. Secondary outcomes will be analyzed descriptively and inferentially without correction, given their exploratory nature. To test the mechanistic hypothesis that ANS modulation and inflammatory/metabolic biomarkers mediate Lo-TENS’s effects on primary outcomes, we will conduct bootstrapped mediation analysis (5,000 resamples) using the PROCESS macro for SPSS 26. For each primary outcome (SBP, FBG, LDL-C), we will test whether the effect of Lo-TENS is mediated by pre-specified mechanistic markers (HF-HRV, norepinephrine, IL-6, and adiponectin), with 95% confidence intervals used to determine significant mediation. The magnitude of the effects will be quantified using partial eta-squared (*η_p_*^2^), with thresholds of 0.01, 0.06, and 0.14 indicating small, medium, and large effect sizes, respectively.

Subsequently, all analyses will be reiterated after the exclusion of drop-outs to conduct a per-protocol analysis.

### 2.9 Ethical considerations

The study protocol is approved by the Ethics Committee of The Second People’s Hospital of Kunming (approval number: 202401011). All participants provide written informed consent. Adverse events (e.g., skin irritation, dizziness) are monitored throughout the study; any serious adverse events are reported to the Ethics Committee and Safety Supervision Committee within 24 h. A Safety Supervision Committee (independent of the study team) oversees participant safety and data integrity. Participants may withdraw from the study at any time without penalty.

## 3 Discussion

### 3.1 Expected findings

This pilot RCT is the first to investigate Lo-TENS as an adjunctive therapy for common multimorbidity in older adults, with a focus on feasibility, efficacy, and mechanisms. We hypothesize that Lo-TENS will be feasible (high recruitment/adherence rates, low attrition/adverse event rates) and effective in reducing SBP, FBG, and LDL-C compared to sham stimulation. We also expect that Lo-TENS will improve ANS function (increased HF-HRV, reduced NE) and modulate inflammatory/metabolic biomarkers (reduced IL-6, increased adiponectin/FGF21), with these markers mediating the intervention’s effects on primary outcomes (5, 22). These findings will provide critical data to inform a fully powered definitive RCT, which is necessary to confirm Lo-TENS’s efficacy and translate it into clinical practice.

### 3.2 Mechanistic implications

The study’s mechanistic focus addresses a key gap in current Lo-TENS research: while prior studies have shown Lo-TENS improves clinical outcomes in single chronic conditions, the pathways linking Lo-TENS to multimorbidity improvement remain unclear (16, 17, 20). By testing mediation by ANS and inflammatory/metabolic markers, we aim to validate the hypothesis that sympathetic over-activation is a unifying target for multimorbidity management (8, 12). If confirmed, this mechanism would support the use of Lo-TENS as a “disease-modifying” adjunctive therapy, rather than just a symptomatic treatment—potentially transforming multimorbidity care in older adults.

### 3.3 Translational potential

Lo-TENS has significant translational potential due to its low cost, non-invasiveness, and ease of administration. If Lo-TENS is found to be feasible and effective in this hospital-based pilot trial, future research will focus on adapting the intervention for community-based self-administration—a critical step for translation to real-world geriatric care (32). This will include evaluating low-cost home Lo-TENS devices, developing caregiver/patient training materials, and testing remote monitoring strategies to ensure consistent stimulation delivery in non-clinical settings. Given Lo-TENS’s low cost and minimal side effects, it has significant potential to reduce polypharmacy burden and improve multimorbidity management in underserved geriatric populations worldwide (6, 7). Additionally, the feasibility data from this pilot will inform the design of larger, multi-center trials to confirm efficacy in diverse older adult populations.

### 3.4 Limitations

This pilot RCT has several limitations that should be acknowledged. First, the small sample size (50 participants) limits the generalizability of efficacy findings; however, this is appropriate for a pilot trial focused on feasibility and effect size estimation (21). Second, the single-center design may limit external validity; future multi-center trials should include diverse clinical settings (e.g., urban/rural hospitals, community health centers) to improve generalizability. Third, the single-blinded design (therapists not blinded) may introduce performance bias; however, we minimized this by ensuring outcome assessors and data analysts are blinded, and we will quantify unblinding bias using a post-intervention survey (30). Fourth, a further limitation is the potential for attrition related to geriatric-specific factors (e.g., mobility limitations, frailty, or polypharmacy-related fatigue), which may impact follow-up completion. To mitigate this, we have implemented flexible intervention scheduling and telephone reminders for follow-up assessments; attrition rates and reasons for dropout will be reported in detail to inform feasibility for future community-based trials. Finally, the 4-week intervention period and 4-week follow-up are short; longer-term trials are needed to assess the sustainability of Lo-TENS’s effects.

### 3.5 Future directions

Based on the pilot’s findings, future research will focus on three key areas: (1) Conducting a fully powered, multi-center RCT to confirm Lo-TENS’s efficacy for multimorbidity in older adults, with a longer intervention/follow-up period (e.g., 12 weeks); (2) Exploring subgroup analyses (e.g., age, number of comorbidities, frailty status) to identify which older adults benefit most from Lo-TENS; (3) Developing and testing a community-based Lo-TENS intervention (self-administered by patients/caregivers) to improve accessibility and scalability (32, 33). Additionally, future studies could explore combining Lo-TENS with other non-pharmacological interventions (e.g., exercise, dietary counseling) to maximize clinical benefits for older adults with multimorbidity.

## Glossary

ANS: Autonomic Nervous System
ABPM: 24-Hour Ambulatory Blood Pressure Monitoring
CRP: C-Reactive Protein
EH: Essential Hypertension
ELISA: Enzyme-Linked Immunosorbent Assay
FBG: Fasting Blood Glucose
FGF21: Fibroblast Growth Factor 21
HF-HRV: High-Frequency Heart Rate Variability
IL-6: Interleukin-6
ITT: Intention-to-Treat
LDL: Low-density lipoprotein
LDL-C: Low-Density Lipoprotein Cholesterol
LF-HRV: Low-Frequency Heart Rate Variability
Lo-TENS: Low-Frequency Transcutaneous Electrical Nerve Stimulation
NE: Norepinephrine
PP: Per-Protocol
RCT: Randomized Controlled Trial
SBP: Systolic Blood Pressure
SF-36: 36-Item Short Form Health Survey
T2DM: Type 2 Diabetes Mellitus
